# Safflower polysaccharide inhibits the development of tongue squamous cell carcinoma

**DOI:** 10.1186/s12957-018-1441-3

**Published:** 2018-08-13

**Authors:** Haiyan Zhou, Jing Yang, Chuhan Zhang, Yuwei Zhang, Rui Wang, Xiao Li, Shuainan Zhang

**Affiliations:** 1 Department of Cleft Palate Speech, The First Affiliated Hospital of Harbin Medical University, Harbin, 150001 People’s Republic of China; 2 Department of Oral and Maxillofacial Surgery, The First Affiliated Hospital of Harbin Medical University, No. 23 Youzheng Road, Nangang District, Harbin, 150001 Heilongjiang Province People’s Republic of China; 3 Department of Basic Medical Science, Heilongjiang University of Chinese Medicine, Harbin, 150040 People’s Republic of China; 4 Department of Pharmacy, Heilongjiang University of Chinese Medicine, Harbin, 150040 People’s Republic of China; 5 Department of Pharmacy, Guiyang University of Chinese Medicine, Guiyang, 550025 People’s Republic of China

**Keywords:** Safflower polysaccharide, Tongue squamous cell carcinoma, Apoptosis, Bcl-2, COX-2, Bax, Cleaved caspase-3

## Abstract

**Background:**

Safflower polysaccharide (SPS) is one of the most important active components of safflower (*Carthamus tinctorius* L.), which has been confirmed to have the immune-regulatory function and antitumor effect. This study aimed to explore the effects of safflower polysaccharide (SPS) on tongue squamous cell carcinoma (TSCC).

**Methods:**

HN-6 cells were treated with 5 μg/mL cisplatin and various concentrations of SPS (0, 0.02, 0.04, 0.08, 0.16, 0.32, 0.64, and 1.28 mg/mL), and cell proliferation was measured. After treatment with 5 μg/mL cisplatin and 0.64 mg/mL SPS, the induction of apoptosis and the protein and mRNA expression of Bax, Bcl-2, COX-2, and cleaved caspase-3 in HN-6 cells were quantified. In addition, HN-6 cells were implanted into mice to establish an in vivo tumor xenograft model. Animals were randomly assigned to three groups: SPS treatment, cisplatin treatment, and the model group (no treatment). The body weight, tumor volume, and tumor weight were measured, and the expression of the above molecules was determined.

**Results:**

SPS treatment (0.02–0.64 mg/mL) for 24–72 h inhibited HN-6 cell proliferation. In addition, 0.64 mg/mL SFP markedly induced apoptosis in HN-6 cells and arrested the cell cycle at the G0/G1 phase. Compared with the control group, the expression of Bcl-2 and COX-2 was markedly reduced by SPS treatment, whereas the expression of Bax and cleaved caspase-3 was increased. Moreover, SPS significantly inhibited the growth of the tumor xenograft, with similar changes in the expression of Bcl-2, COX-2, Bax, and cleaved caspase-3 in the tumor xenograft to the in vitro analysis.

**Conclusions:**

Our results indicated that SPS may inhibit TSCC development through regulation of Bcl-2, COX-2, Bax, and cleaved caspase-3 expression.

## Background

Tongue squamous cell carcinoma (TSCC) is a primary malignant tumor of the tongue, with the highest incidence rate (approximately 39.95%) among oral cancers [1]. With early detection, TSCC can be cured with proper treatment; however, after tumor metastasis, the 5-year overall survival rate is below 50% [2–4]. Therefore, the research and development of effective drugs and methods for the treatment of TSCC would have far-reaching consequences.

Safflower (*Carthamus tinctorius* L.) is a herbaceous plant of the Asteraceae family, containing various active constituents, including flavonoids, quinochalcones, alkaloids, and safflower polysaccharides (SPS) [5]. Safflower exerts various biological effects, including antioxidant [6], anti-inflammatory [7], and antibacterial [8] activities, and is reported to be beneficial for the improvement of acute cerebral infarction [9] and ischemic stroke [10]. SPS is one of the most important active components of safflower, and accumulating evidences have supported the immuno-regulatory function and antitumor effect of SPS [11–13]. In breast cancer, SPS is shown to inhibit the MCF-7 cell proliferation and metastasis [14]. SPS is also found to inhibit proliferation of human hepatic cancer SMMC-7721 cells through the regulation of the expression of cell cycle-related genes [15]. Moreover, SPS is confirmed to affect cell growth and apoptosis in non-small cell lung cancer [16], gastric cancer [17–19], and colorectal cancer [20]. However, the role of SPS in the development of TSCC remains unexplored.

In this study, we detected the effect of SPS on HN-6 cell proliferation and apoptosis. Moreover, HN-6 cells were implanted into mice to establish an in vivo tumor xenograft model for the assessment of the effect of SPS on tumor growth. The present study investigated the roles and regulatory mechanism of SPS in TSCC to provide new strategies for TSCC therapy.

## Methods

### SPS preparation

The crude drug containing SPS was purchased from Shiyitang Co., Ltd. (Harbin, PR China), and voucher specimens (No. HLJ-2015008) were deposited at College of Basic Medical Science, Heilongjiang University of Chinese Medicine. The crude drug was dried at 60 °C in a vacuum oven for 24 h and extracted four times in boiling water with agitation for 1 h. The extracts were filtered, concentrated, and precipitated with four volumes of 95% ethanol at 4 °C for 24 h. The mixture was centrifuged, and the sediment was dried at 60 °C in a vacuum oven. The protein contaminants were extracted with Sevage reagent (a 4:1 (*v*/*v*) mixture of chloroform to *n*-butyl alcohol) and removed by centrifugation; this process was repeated 10 times. The water phase was then precipitated in four volumes of 95% ethanol. The sediments were oven-dried at 60 °C to produce SPS, a light-yellow powder, at yield of 0.382% (*w*/*w*). SPS was composed of d-glucose in a weight ratio of 97.06%.

### Cell culture

The TSCC cell line, HN-6, was obtained from the Laboratory of Oncological Biology of the Ninth Hospital Affiliated to Shanghai Jiao Tong University (Shanghai, PR China). HN-6 cells were then maintained in Dulbecco’s modified Eagle medium (DMEM; pH 7.2; Sigma-Aldrich, Shanghai, PR China) supplemented with 10% fetal bovine serum (FBS; Sigma-Aldrich) in a 37 °C incubator with a humidified atmosphere of 5% CO_2_.

### CCK-8 assay

For the detection of cell proliferation, the Cell Counting Kit-8 (CCK-8) kit (Dojindo, Shanghai, PR China) was used in accordance with the manufacturer’s instructions. HN-6 cells were seeded in a 96-well plate (6.0 × 10^6^ cells/well) and cultured in DMEM supplemented with 10% FBS at 37 °C for 24 h. SPS and cisplatin (Qilu Pharmaceutical Co., Ltd., Jinan, PR China) were dissolved in DMEM prior to use. Subsequently, 5 μg/mL cisplatin and various concentrations of SPS (0, 0.02, 0.04, 0.08, 0.16, 0.32, 0.64, and 1.28 mg/mL) were added to each well of HN-6 cells separately and incubated at 37 °C for 24, 48, and 72 h. Optical density (OD) at 450 nm was determined. The inhibitory rate (IR) of cell proliferation was calculated from the following equation: IR = 1 − (OD of treated group/OD of control group) × 100%.

### Apoptosis analysis

Apoptosis was analyzed by acridine orange/ethidium bromide (AO/EB) double staining. Briefly, HN-6 cells were seeded in a 6-well plate and incubated with DMEM supplemented with 10% FBS at 37 °C for 48 h. Subsequently, 5 μg/mL cisplatin and 0.64 mg/mL SPS were added separately and incubated with the cells for 24, 48, and 72 h. After this, 5 μL dye mixture (500 mg/mL AO and 500 mg/mL EB in distilled water) was added to each well. Cell apoptosis was then examined by an inverted phase-contrast microscope (Olympus IX70, Hamburg, Germany) at × 400 magnification.

### Cell cycle analysis

Cell cycle analysis was performed using a Cell Cycle Detection Kit (NanJing KeyGen Biotech Co., Ltd., Nanjing, JiangSu, China). Briefly, HN-6 cells were seeded in a 6-well plate and incubated with DMEM supplemented with 10% FBS at 37 °C for 48 h. Then, 5 μg/mL cisplatin and 0.64 mg/mL SPS were added to each well and incubated with the cells for 48 h. The cells were harvested, fixed with 75% ice-cold ethanol, and stained with 400 μL propidium iodide (PI) for 45 min in the dark. The cell cycle analysis after different drug treatments was then conducted using a FACSCalibur flow cytometer (BD, USA).

### qRT-PCR

Total RNA was extracted using the Trizol kit (Invitrogen, Carlsbad, CA, USA), reverse transcription was then performed using an M-MLV RTase kit (Promega, USA), and qRT-PCR was then performed using One Step SYBR®PrimeScript® RT-PCR Kit (Takara, PR China) and an ABI-7500 PCR machine (Applied Biosystems, USA). The amplification procedure comprised 95 °C for 15 s, followed by 40 cycles of 95 °C for 10 s and 60 °C for 30 s. The forward and reverse primer sequences, respectively, for the amplification of targets were as follows: Bax, 5′-GGCCCTTTTGCTTCAGGGTT-3′ and 5′-GGAAAAAGACCTCTCGGGGG-3′; Bcl-2, 5′-CTTTGAGTTCGGTGGGGTCA-3′ and 5′-GGGCCGTACAGTTCCACAAA-3′; COX-2, 5′-TTTGCATTCTTTGCCCGC-3′ and 5′-GGGAGGATACATCTCTCCATCAAT-3′; cleaved caspase-3, 5′-AGCAATAAATGAATGGGCTGAG-3′ and 5′-GTATGGAGAAATGGGCTGTAGG-3′; and GAPDH, 5′-CGCTGAGTACGTCGTGGAGTC-3′ and 5′-GCTGATGATCTTGAGGCTGTTGTC-3′. The relative expression of these target genes were normalized to GAPDH and then calculated using the 2^-ΔΔCT^ method.

### Western blot analysis

Total protein was extracted in lysis buffer containing PMSF (Pierce; Rockford, IL). The proteins were separated in a 10% SDS-PAGE and immunoblotted onto polyvinylidene fluoride (PVDF) membranes (Millipore, Boston, USA). Non-specific binding to the membranes was blocked by incubation with 5% nonfat dried milk for 1–2 h, and the membranes were probed separately with rabbit anti-human Bax, Bcl-2, COX-2, cleaved caspase-3, and GAPDH antibodies (1:100, Invitrogen) overnight at 4 °C, followed incubation with the appropriate horseradish peroxidase-conjugated secondary antibody (1:10000, Invitrogen) for 1 h; GAPDH was used as the internal control. The protein bands were visualized by the application of 4-chloronaphthol (Sigma-Aldrich) and analyzed by Gel-Pro 4.0 software (Media Cybernetics, Inc., USA).

### In vivo tumor xenograft model

Twenty female BALB/cnu/nu nude mice (4–6 weeks old) were purchased from Beijing Vital River Laboratory Animal Technology Co., Ltd. (Beijing, China). These mice were housed in a room at constant temperature (27 °C ± 1 °C) and humidity (50% ± 10%) and a 12-h light/dark cycle for 1 week. HN-6 cells (4 × 10^7^) were subcutaneously injected into the flanks of mice to establish the in vivo tumor xenograft model. When the volumes of the xenografts reached 100–300 mm^3^, the animals were randomly divided into three groups: SPS (*n* = 8), cisplatin (*n* = 8), and model (*n* = 4). Throughout the 15 days of treatment, the mice in the SPS group were injected with 40 mg/kg SPS once a day, and the mice in cisplatin and model groups were injected with 0.8 mL/day cisplatin and normal saline every 3 days, respectively. The animal experiments were approved by the Animal Care and Use Committee of China Medical University (Taichung, Taiwan).

During treatment, the body weight of the tumor-bearing mice and the tumor size (length and width) were measured every 3 days. The tumor volume was estimated from the following equation: tumor volume = 0.5*ab*^2^, where *a* is the length of the tumor and *b* is the width of the tumor. The animals were sacrificed at the end of the study, and the tumors were removed and weighed. Tumor xenografts were used for the analysis of the expression of COX-2, Bcl-2, Bax, and cleaved caspase-3 by qRT-PCR and western blot analysis.

### Statistical analysis

All measurement data from multiple experiments were presented as the mean ± standard deviation. One-way ANOVA was performed to analyze the significance of differences among groups, followed by a Tukey post hoc test for further between-group comparisons. Statistical software SPSS 17.0 (SPSS Inc., Chicago, IL, USA) was applied, and statistical significance was accepted at *P* < 0.05.

## Results

### SPS inhibited HN-6 cell proliferation

The effect of SPS on HN-6 cell proliferation was evaluated by a CCK8 assay. The results indicated that SPS inhibited HN-6 cell proliferation in a dose- and time-dependent manner within a certain dose range (0.02–0.64 mg/mL) and time (24–72 h) (Table 1). Among these concentrations of SPS (0.02–1.28 mg/mL), 0.64 mg/mL SPS exhibited the strongest IR on HN-6 cell proliferation at different time points (Table 1), and this concentration was therefore selected for subsequent experiments.

**Table 1:** The effect of SPS on HN-6 cell proliferation

Group	Concentration (mg/mL)	OD value (24 h)	IR%	OD value (48 h)	IR%	OD value (72 h)	IR%
SPS	0.02	0.816 ± 0.028	1.73	0.798 ± 0.033*	10.82	0.649 ± 0.023*	27.71
	0.04	0.811 ± 0.054	4.74	0.760 ± 0.022*	16.24	0.643 ± 0.036*	31.43
	0.08	0.772 ± 0.033	12.41	0.659 ± 0.012*	20.18	0.622 ± 0.019*	35.33
	0.16	0.677 ± 0.028	18.13	0.619 ± 0.015*	35.41	0.582 ± 0.052*	38.62
	0.32	0.628 ± 0.056	33.33	0.496 ± 0.033*	42.53	0.321 ± 0.031*	47.78
	0.64	0.488 ± 0.027	46.72	0.309 ± 0.031*	51.29	0.266 ± 0.058**	64.95
	1.28	0.53 ± 0.081	42.63	0.451 ± 0.042*	48.12	0.306 ± 0.036*	56.32
Cisplatin	5 × 10^-3^	0.461 ± 0.034	58.66	0.283 ± 0.011**	75.59	0.308 ± 0.032**	72.09
Control	0.00	0.822 ± 0.021	-	0.850 ± 0.023	-	0.996 ± 0.012	-

*SPS* safflower polysaccharide, *OD* optical density, *IR* inhibitory rate. *P <0.05 and **P < 0.01

### SPS induced the apoptosis of HN-6 cells

**Fig. 1:**
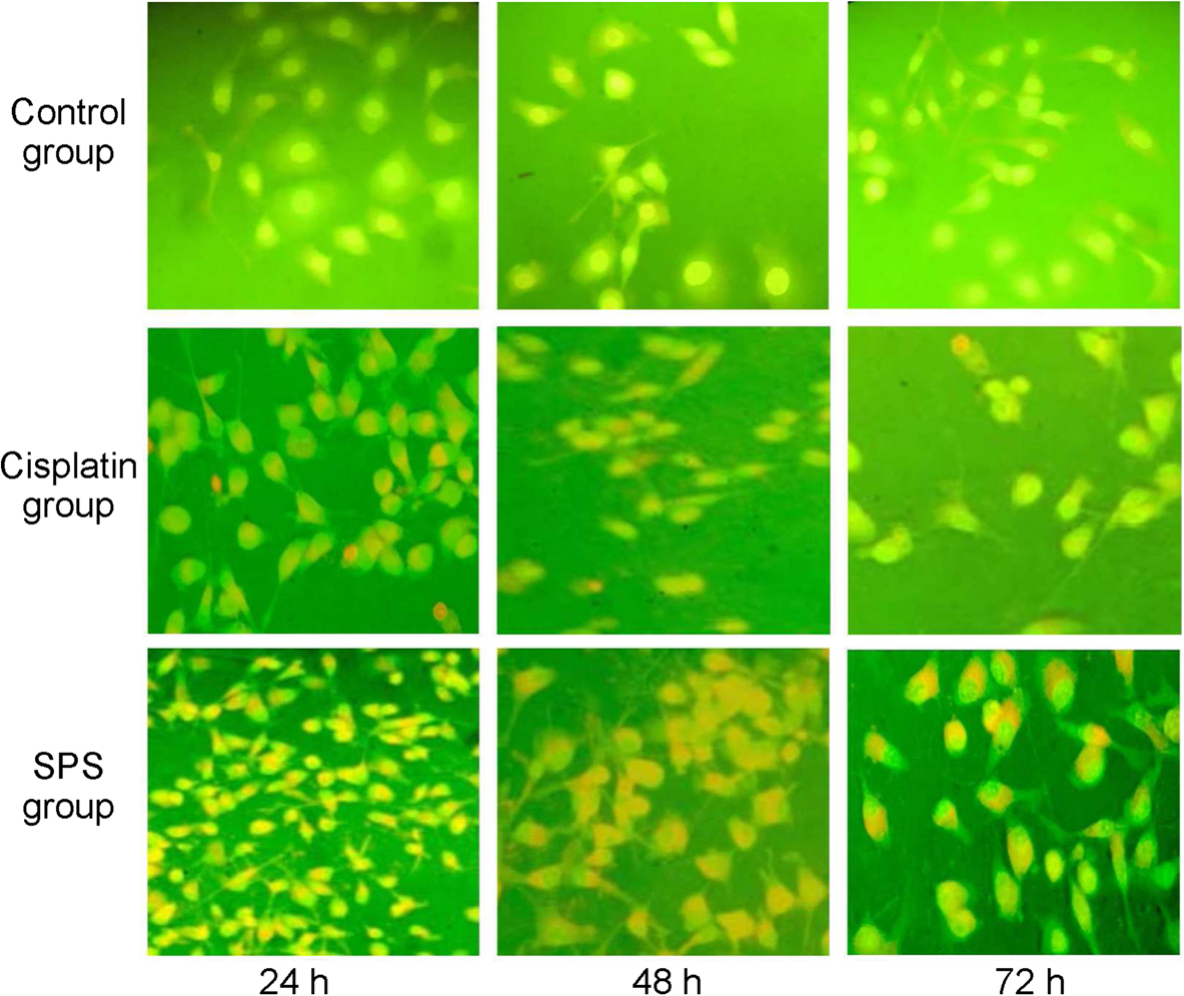
Acridine orange/ethidium bromide (AO/EB) double staining (× 400) was used to detect the apoptosis of HN-6 cells in the control-, 5 μg/mL cisplatin-, and 0.64 mg/mL SPS-treated groups

AO/EB double staining showed that the morphologies of HN-6 cells in the control group had intact structure and green-stained nuclei (Fig. 1). After treatment with cisplatin or 0.64 mg/mL SPS, shrinkage, chromatin condensation, membrane blebbing, and the formation of apoptotic bodies were identified in HN-6 cells (Fig. 1), indicating that SPS induced apoptosis in HN-6 cells.

### SPS arrested cell cycle in the G0/G1 phase

The effect of SPS on the cell cycle was also examined. Compared with the control group, the percentage of HN-6 cells in the G0/G1 phase was significantly increased after treatment with 0.64 mg/mL SPS for 48 h, whereas the percentage of HN-6 cells in the G2/M phase was markedly decreased (Table 2), which indicated that SPS arrested the cell cycle of HN-6 cells in the G0/G1 phase. The percentage of HN-6 cells in different cell cycle stages was not significantly different in the control and cisplatin groups (Table 2).

**Table 2:** The effect of 0.64 mg/mL SPS on HN-6 cell cycle

Groups	G0/G1 (%)	S	G2/M
Control	10.71	52.42	36.87
SPS	25.76*	50.49	23.75*
Cisplatin	14.51	47.99	37.50

*SPS* safflower polysaccharide. *P < 0.05

### Analysis of mRNA and protein expression of Bcl-2, COX-2, Bax, and cleaved caspase-3 in HN-6 cells after treatment

To investigate the regulatory mechanism of SPS, the mRNA and protein expression of Bcl-2, COX-2, Bax, and cleaved caspase-3 were detected. The results showed that, compared with the control group, the expression of Bcl-2 and COX-2 mRNA and protein was significantly decreased after SPS or cisplatin treatment in a time-dependent manner, whereas that of Bax and cleaved caspase-3 was obviously increased (Tables 3 and 4).

**Table 3:** The relative expression of target mRNAs in the control, cisplatin, and 0.64 mg/mL SPS groups

Groups	Bcl-2	COX-2	Bax	Cleaved caspase-3
Control (24 h)	0.713 ± 0.031	0.638 ± 0.048	0.185 ± 0.043	0.262 ± 0.081
Control (48 h)	0.720 ± 0.196	0.592 ± 0.005	0.166 ± 0.121	0.321 ± 0.021
Control (72 h)	0.693 ± 0.232	0.799 ± 0.923	0.203 ± 0.056	0.301 ± 0.044
Cisplatin (24 h)	0.585 ± 0.055	0.513 ± 0.023*	0.481 ± 0.011*	0.477 ± 0.021*
Cisplatin (48 h)	0.451 ± 0.060*	0.416 ± 0.019*	0.566 ± 0.035*	0.681 ± 0.046*
Cisplatin (72 h)	0.281 ± 0.026*	0.264 ± 0.032*	0.762 ± 0.055*	0.872 ± 0.065*
SPS (24 h)	0.423 ± 0.055*	0.408 ± 0.023*	0.314 ± 0.011*	0.585 ± 0.034*
SPS (48 h)	0.345 ± 0.060*	0.222 ± 0.019*	0.426 ± 0.035	0.822 ± 0.081*
SPS (72 h)	0.201 ± 0.026*	0.118 ± 0.032*	0.582 ± 0.055*	1.031 ± 0.092*

*SPS* safflower polysaccharide. *P < 0.05 compared with the control group

**Table 4:** The relative expression of the target proteins in the control, cisplatin, and 0.64 mg/mL SPS groups

Groups	Bcl-2	COX-2	Bax	Cleaved caspase-3
Control (24 h)	0.723 ± 0.121	0.918 ± 0.095	0.282 ± 0.164	0.464 ± 0.091
Control (48 h)	0.719 ± 0.196	0.922 ± 0.132	0.283 ± 0.053	0.460 ± 0.021
Control (72 h)	0.733 ± 0.198	0.899 ± 0.210	0.290 ± 0.026	0.465 ± 0.018
Cisplatin (24 h)	0.442 ± 0.113*	0.661 ± 0.066*	0676 ± 0.103*	0.679 ± 0.093*
Cisplatin (48 h)	0.393 ± 0.045*	0.458 ± 0.072*	0.867 ± 0.064*	0.863 ± 0.123*
Cisplatin (72 h)	0.107 ± 0.062	0.237 ± 0.069	0.916 ± 0.093*	0.991 ± 0.133*
SPS (24 h)	0.513 ± 0.101*	0.795 ± 0.135*	0.740 ± 0.0858	0.602 ± 0.076*
SPS (48 h)	0.392 ± 0.083*	0.590 ± 0.099*	0.920 ± 0.083*	0.764 ± 0.063*
SPS (72 h)	0.206 ± 0.106*	0.435 ± 0.021*	1.832 ± 0.925*	0.948 ± 0.089*

*SPS* safflower polysaccharide. *P < 0.05 compared with the control group

### SPS inhibited the growth of tumor xenograft

**Fig. 2:**
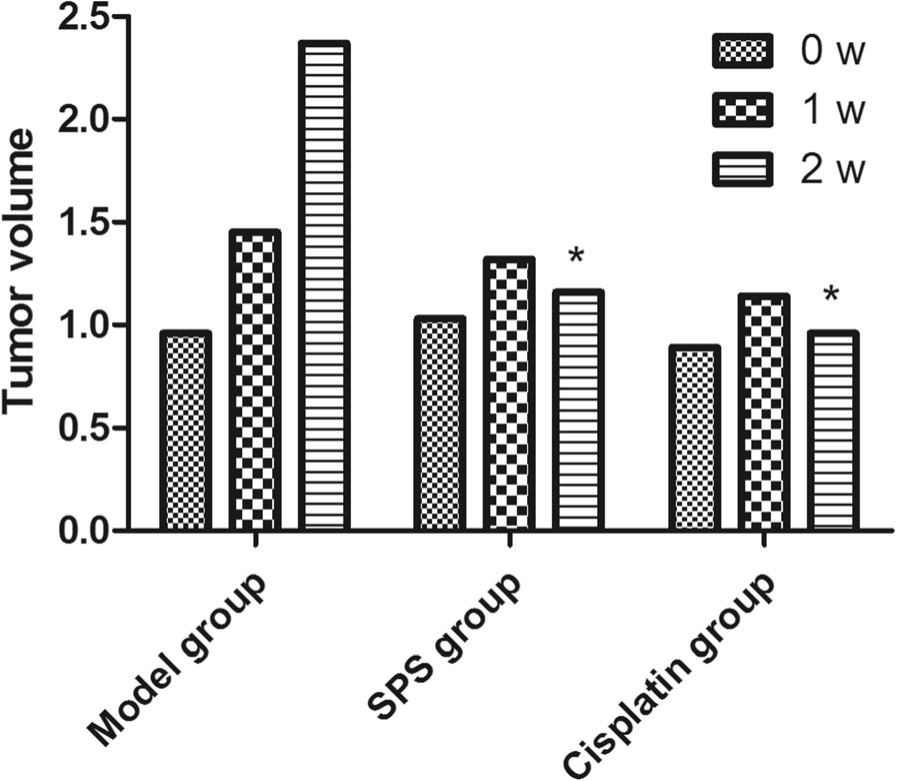
The tumor volume of mice in the model, SPS, and cisplatin groups after intervention for 1 and 2 weeks. The data are presented as the mean ± standard deviation. *P < 0.05 compared with the model group after the same treatment time

The in vivo tumor xenograft model was established to explore the effects of SPS. The tumor weights in the model, SPS, and cisplatin groups were 2.236 ± 0.063, 1.145 ± 0.210, and 0.963 ± 0.049 g, respectively. Moreover, compared with the model group, the tumor volume of the SPS and cisplatin groups were markedly decreased after 2 weeks of intervention (*P* < 0.05, Fig. 2). However, there were no significant differences in the body weight of mice in the different groups (data not shown).

### Analysis of mRNA and protein expression of Bcl-2, COX-2, Bax, and cleaved caspase-3 in tumor xenograft model after different treatments

**Fig. 3:**
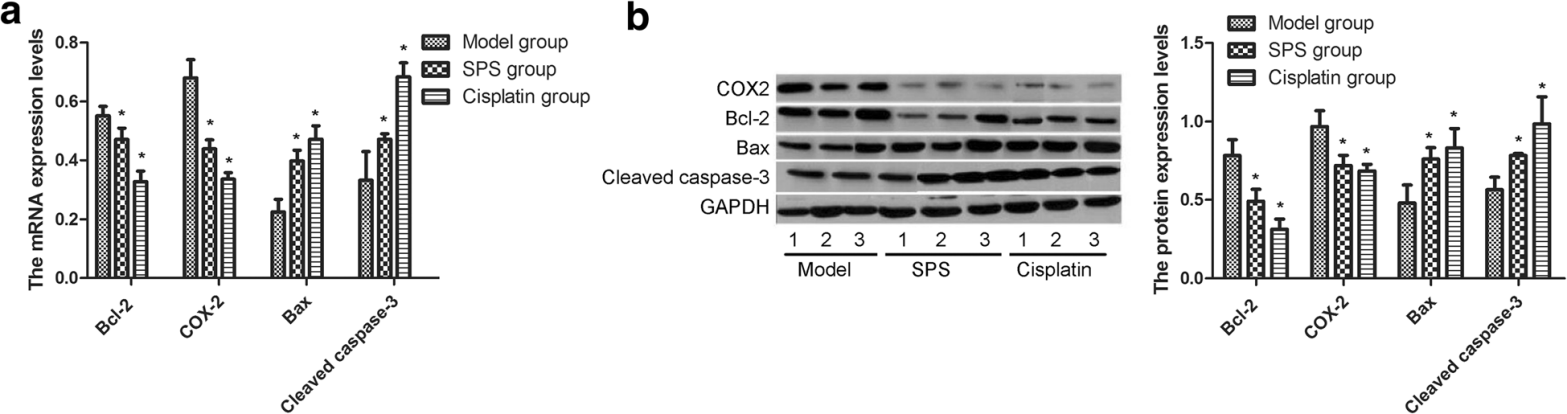
Analysis of the mRNA and protein expression of Bcl-2, COX-2, Bax, and cleaved caspase-3 in tumor xenograft model after different interventions. **a**: The mRNA expression of Bcl-2, COX-2, Bax and cleaved caspase-3; **b**: The protein expression of Bcl-2, COX-2, Bax and cleaved caspase-3. The data are presented as the mean ± standard deviation. *P < 0.05 compared with the model group

Consistent with the expression changes in HN-6 cells after different treatment, Bcl-2 and COX-2 expression in the tumor xenografts from the SPS or cisplatin groups was significantly lower than those from the model group, and the expression of Bax and cleaved caspase-3 was increased (Fig. 3).

## Discussion

The present study illustrated that SPS markedly inhibited HN-6 cell proliferation, induced apoptosis, and arrested the cell cycle of HC-6 cells in the G0/G1 phase. Bcl-2 and COX-2 expression was significantly decreased after SPS treatment, whereas Bax and cleaved caspase-3 was significantly increased. Moreover, SPS significantly inhibited growth of the in vivo tumor xenografts, and the changes in the expression of the above molecules in tumor xenograft model after SPS intervention were consistent with previous results.

Apoptosis is an important mechanism involved in cancer progression, involving a series of active death process after the stimulation of many types of death signals [21, 22]. Bcl-2 families, such as Bax and Bcl-2, are regarded as a key mediator of cell apoptosis [23]. The ratio of Bcl-2 to Bax is shown to affect cellular sensitivity to the apoptotic signals. A previous study has confirmed that the mechanism of cantharidin in the promotion of apoptosis in TSCC may be associated with the suppression of the Bcl-2/Bax signaling pathway [24]. Moreover, Bcl-2 inhibition and Bax activation are thought to be promising approaches for cancer therapy [25, 26]. Notably, safflower injection resulted in an increase in the Bax/Bcl-2 ratio [27], which prompted us to speculate that SPS may inhibit TSCC development through the regulation of the Bcl-2/Bax expression ratio. In addition, caspase-3 is also considered to be a key mediator of mitochondrial apoptosis [28]. Cleaved caspase-3 can induce apoptosis through blocking the contact between the cell and its surroundings [29, 30]. Importantly, the activation of caspase-3 can induce apoptosis in TSCC [31]. In this study, we found that SPS induced apoptosis in HN-6 cells. Moreover, the Bax/Bcl-2 ratio and the expression of cleaved caspase-3 were increased after SPS treatment. Therefore, SPS may promote cell apoptosis in TSCC through an increase in the Bax/Bcl-2 ratio and the expression of cleaved caspase-3.

Furthermore, we also found that the expression of COX-2 was significantly decreased after SPS treatment. COX-2 is an inducible enzyme implicated in the transformation of arachidonic acid to prostaglandin and other eicosanoids [32]. An accumulation of evidence supports the overexpression of COX-2 in various tumor tissues and cells and a close relationship with tumor development [33]. Cao et al. demonstrated that miR-26b regulated cell proliferation and metastasis in TSCC through the regulation of COX-2 [34]. Moreover, COX-2 inhibition suppresses angiogenesis and tumor growth, potentiating antiangiogenic cancer therapy [35]. Consistent with a previous study, in which the dried aqueous extracts of safflower petal attenuated COX-2 protein expression and protected against lipopolysaccharide-induced inflammation [36], we found that SPS treatment resulted in a decrease in the expression of COX-2. Given the key role of COX-2 in tumor development, we speculated that SPS may inhibit TSCC development through a decrease in the expression of COX-2.

## Conclusions

In conclusion, our results indicated that SPS may inhibit TSCC development through the regulation of the expression of Bcl-2, COX-2, Bax, and cleaved caspase-3. However, as this study is preliminary, further experiments are required to explore the possible mechanism of SPS in the prevention of TSCC development.

## References

[1] Li H, Zhang Y, Chen SW, Li FJ, Zhuang SM, Wang LP, Zhang J, Song M (2014). Prognostic significance of Flotillin1 expression in clinically N0 tongue squamous cell cancer. Int J Clin Exp Pathol.

[2] Cannon TL, Lai DW, Hirsch D, Delacure M, Downey A, Kerr AR, Bannan M, Andreopoulou E, Safra T, Muggia F (2012). Squamous cell carcinoma of the oral cavity in nonsmoking women: a new and unusual complication of chemotherapy for recurrent ovarian cancer?. Oncologist.

[3] Dibble EH, Alvarez ACL, Truong M-T, Mercier G, Cook EF, Subramaniam RM (2012). 18F-FDG metabolic tumor volume and total glycolytic activity of oral cavity and oropharyngeal squamous cell cancer: adding value to clinical staging. J Nucl Med.

[4] Trotta BM, Pease CS, Rasamny JJ, Raghavan P, Mukherjee S (2011). Oral cavity and oropharyngeal squamous cell cancer: key imaging findings for staging and treatment planning. Radiographics.

[5] Wakabayashi T, Hirokawa S, Yamauchi N, Kataoka T, Woo J-T, Nagai K (1997). Immunomodulating activities of polysaccharide fractions from dried safflower petals. Cytotechnology.

[6] Ali Sahari M, Morovati N, Barzegar M, Asgari S (2014). Physicochemical and antioxidant characteristics of safflower seed oil. Curr Nutr Food Sci.

[7] Toma W, Guimarães LL, Brito AR, Santos AR, Cortez FS, Pusceddu FH, Cesar A, Júnior SL, Pacheco MT, Pereira CD (2014). Safflower oil: an integrated assessment of phytochemistry, antiulcerogenic activity, and rodent and environmental toxicity. Revista Brasileira de Farmacognosia.

[8] Sabah FS, Saleh AA. Evaluation of antibacterial activity of flavonoid and oil extracts from safflower (Carthamus tinctorius L). Evaluation. 2015;5:41–44.

[9] Li L-J, Li Y-M, Qiao B-Y, Jiang S, Li X, Du H-M, Han P-C, Shi J (2015). The value of safflower yellow injection for the treatment of acute cerebral infarction: a randomized controlled trial. Evid Based Complement Alternat Med.

[10] Fan S, Lin N, Shan G, Zuo P, Cui L (2014). Safflower yellow for acute ischemic stroke: a systematic review of randomized controlled trials. Complement Ther Med.

[11] Ando I, Tsukumo Y, Wakabayashi T, Akashi S, Miyake K, Kataoka T, Nagai K (2002). Safflower polysaccharides activate the transcription factor NF-κB via Toll-like receptor 4 and induce cytokine production by macrophages. Int Immunopharmacol.

[12] Shi X, Ruan D, Wang Y, Ma L, Li M (2010). Anti-tumor activity of safflower polysaccharide (SPS) and effect on cytotoxicity of CTL cells, NK cells of T739 lung cancer in mice. Zhongguo Zhong yao za zhi= Zhongguo zhongyao zazhi=China journal of Chinese materia medica.

[13] Xi SY, Zhang Q, Wang C, Zhang JJ, Gao XM (2008). Discussion of safflower inhibiting tumor in application and its mechanism of action. Ch inese Archives of Traditional Chinese Medicine.

[14] Luo Z, Zeng H, Ye Y, Liu L, Li S, Zhang J, Luo R (2015). Safflower polysaccharide inhibits the proliferation and metastasis of MCF-7 breast cancer cell. Mol Med Rep.

[15] SUN Y, YANG J, ZHANG Q-q, WANG X, XU F, LI M-z, WANG Y-x: Mechanism investigation of cell cycle arrest in hepatic cancer cell induced by safflower polysaccharide Chinese J Experimental Traditional Medical Formulae 2014; 13**:**046.

[16] Li J-Y, Yu J, Du X-S, Zhang H-M, Wang B, Guo H, Bai J, Wang J-H, Liu A, Wang Y-L (2016). Safflower polysaccharide induces NSCLC cell apoptosis by inhibition of the Akt pathway. Oncol Rep.

[17] Xinbo M, Zhenzuo Z, Rufei G, Xue-kui S, Lin S, An-wen Z, Hai-guang S, Shen Y, Ya-xian W (2012). The pilot study on the inhibition of safflower polysaccharide to human gastric carcinoma cell line SGCY7901. Guangxi Medical J.

[18] Wang T, Shi X, Sun Y, WANG Y-x: Experimental study on morphologic effect of safflower polysaccharide on the apoptosis of gastric carcinoma cell line SGC-7901. Information Traditional Chinese Medicine 2015; 32**:**19–21.

[19] Tao J, Li QW, Shi XK, Liang Y, Wang YX: Safflower polysaccharide inhibit PI3K/Akt signaling pathway induces apoptosis of human gastric cancer cells. Practical oncology Journal 2012; 26**:**119–124.

[20] Liang A, Jianghong Z, Taijun Z, Xiaoqing L, Qiong Z, Jun C. Analysis of the inhibitory effect of safflower polysaccharide on HT29 colorectal cancer cell proliferation and its relevant mechanism. Biomed Res. 2017;28:2966–70.

[21] Porter AG, Janicke RU (1999). Emerging roles of caspase-3 in apoptosis. Cell Death Differ.

[22] Hockenbery DM, Oltvai ZN, Yin XM, Milliman CL, Korsmeyer SJ (1993). Bcl-2 functions in an antioxidant pathway to prevent apoptosis. Cell.

[23] Risso A, Mercuri F, Quagliaro L, Damante G, Ceriello A (2001). Intermittent high glucose enhances apoptosis in human umbilical vein endothelial cells in culture. Am J Physiol Endocrinol Metab.

[24] Tian X, Zeng G, Li X, Wu Z, Wang L (2015). Cantharidin inhibits cell proliferation and promotes apoptosis in tongue squamous cell carcinoma through suppression of miR-214 and regulation of p53 and Bcl-2/Bax. Oncol Rep.

[25] Xin M, Li R, Xie M, Park D, Owonikoko TK, Sica GL, Corsino PE, Zhou J, Ding C, White MA (2014). Small-molecule Bax agonists for cancer therapy. Nat Commun.

[26] Souers AJ, Leverson JD, Boghaert ER, Ackler SL, Catron ND, Chen J, Dayton BD, Ding H, Enschede SH, Fairbrother WJ (2013). ABT-199, a potent and selective BCL-2 inhibitor, achieves antitumor activity while sparing platelets. Nat Med.

[27] LIU J, XU X f, YANG W j, Guo C y: Effect of safflower injection on proliferation, apoptosis, and expression of bcl-2/bax gene in hepatic stellate cell in vitro [J]. Chinese Traditional and Herbal Drugs 2009; 8**:**030.

[28] Lakhani SA, Masud A, Kuida K, Porter GA, Booth CJ, Mehal WZ, Inayat I, Flavell RA (2006). Caspases 3 and 7: key mediators of mitochondrial events of apoptosis. Science.

[29] Pan JA, Ullman E, Dou Z, Zong WX (2011). Inhibition of protein degradation induces apoptosis through a microtubule-associated protein 1 light chain 3-mediated activation of caspase-8 at intracellular membranes. Mol Cell Biol.

[30] Huang Q, Li F, Liu X, Li W, Shi W, Liu FF, O'Sullivan B, He Z, Peng Y, Tan AC (2011). Caspase 3-mediated stimulation of tumor cell repopulation during cancer radiotherapy. Nat Med.

[31] Coutinho-Camillo CM, Lourenço SV, Nishimoto IN, Kowalski LP, Soares FA (2011). Caspase expression in oral squamous cell carcinoma. Head & neck.

[32] Khan Z, Khan N, P Tiwari R, K Sah N, Prasad G, S Bisen P (2011). Biology of Cox-2: an application in cancer therapeutics. Curr Drug Targets.

[33] Song X, Lin HP, Johnson AJ, Tseng PH, Yang YT, Kulp SK, Chen CS (2002). Cyclooxygenase-2, player or spectator in cyclooxygenase-2 inhibitor-induced apoptosis in prostate cancer cells. J Natl Cancer Inst.

[34] Cao J, Guo T, Dong Q, Zhang J, Li Y (2015). miR-26b is downregulated in human tongue squamous cell carcinoma and regulates cell proliferation and metastasis through a COX-2-dependent mechanism. Oncol Rep.

[35] Xu L, Stevens J, Hilton MB, Seaman S, Conrads TP, Veenstra TD, Logsdon D, Morris H, Swing DA, Patel NL (2014). COX-2 inhibition potentiates antiangiogenic cancer therapy and prevents metastasis in preclinical models. Sci Transl Med.

[36] Wang CC, Choy CS, Liu YH, Cheah KP, Li JS, Wang JTJ, Yu WY, Lin CW, Cheng HW, Hu CM (2011). Protective effect of dried safflower petal aqueous extract and its main constituent, carthamus yellow, against lipopolysaccharide-induced inflammation in RAW264. 7 macrophages. J Sci Food Agric.

